# Book Reviews

**Published:** 1871-09

**Authors:** 


### BIBLIOGRAPHICAL.

*Taking Impressions of the Mouth.*—A work with this title has recently been issued by James W. White, as editor and Samuel S. White publisher, to which is appended a Chapter on "Porcelain Teeth," from the new edition of Harris's Principles and Practice of Dentistry, in which will be found a great deal of information interesting to many of the members of our profession.

The work is written in a clear, comprehensive style and enters into the subject very minutely, describing a number of different processes by which this important operation of dental mechanism can be accomplished. It is profusely illustrated, and appears in a style which is suggestive of the energy and good taste of the publisher. It contains in the form of a finely executed lithograph, a quotation from Josiah Wedgwood, which may be readily removed and framed for the benefit of patients in the dental office, and which will prove servicable as a forcible argument against quackery and cheap dentistry.

We cheerfully recommend this work to all dental practitioners.

*A Review of Darwin's Theory of the Origin and Development of Man.*—By James D. Hunter, M. D. Published by D. Appleton & Co., New York. The Editor of this work, which is well written, while he admits that the strongest arguments of Mr. Darwin, based on the well known and remarkable similarity between man and the lower animals in embryonic developments, upon similarity of structure and constitution, and upon the rudiments which man retains of extinct organs found in perfection lower in the scale, are striking facts that cannot be ignored, yet does not find it so easy to believe that all the vast difference intellectually and morally, between man and his brute brethren can be accounted for by development alone, as Mr. Darwin asserts.

*Plastics and Orthopedics.*—A report from the Transactions of the Illinois State Medical Society for 1871. By David Prince, M. D. This report is a full and comprehensive one on the principles, expedients and specialists of *Plastics* and the different operations pertaining to Orthopedics, and is illustrated by twenty-eight wood cuts.

*New Remedies.*—A quarterly retrospect of Therapeutics, Pharmacy and Allied Subjects. Edited by Horatio C. Wood, Jr., M. D., Professor of Medical Botany. University of Penn. etc. The July No. contains much useful information under the respective headings of Therapeutics, Materia Medica, Toxicology, Prescriptions and Formulas, General Receipts, Druggists Sundries and Manufactures. Published by Wm. Wood & Co., New York. Terms $2.00 per annum.

---

